# Monitoring Temporal Changes in the Specificity of an Oral HIV Test: A Novel Application for Use in Postmarketing Surveillance

**DOI:** 10.1371/journal.pone.0012231

**Published:** 2010-08-25

**Authors:** Joseph R. Egger, Kevin J. Konty, Jessica M. Borrelli, Julia Cummiskey, Susan Blank

**Affiliations:** 1 Bureau of Epidemiology Services, New York City Department of Health and Mental Hygiene, New York, New York, United States of America; 2 Bureau of STD Prevention and Control, New York City Department of Health and Mental Hygiene, New York, New York, United States of America; 3 Division of STD Prevention, U.S. Centers for Disease Control and Prevention, Atlanta, Georgia, United States of America; McGill University Health Center, Canada

## Abstract

**Background:**

Postmarketing surveillance is routinely conducted to monitor performance of pharmaceuticals and testing devices in the marketplace. However, these surveillance methods are often done retrospectively and, as a result, are not designed to detect issues with performance in real-time.

**Methods and Findings:**

Using HIV antibody screening test data from New York City STD clinics, we developed a formal, statistical method of prospectively detecting temporal clusters of poor performance of a screening test. From 2005 to 2008, New York City, as well as other states, observed unexpectedly high false-positive (FP) rates in an oral fluid-based rapid test used for screening HIV. We attempted to formally assess whether the performance of this HIV screening test statistically deviated from both local expectation and the manufacturer's claim for the test. Results indicate that there were two significant temporal clusters in the FP rate of the oral HIV test, both of which exceeded the manufacturer's upper limit of the 95% CI for the product. Furthermore, the FP rate of the test varied significantly by both STD clinic and test lot, though not by test operator.

**Conclusions:**

Continuous monitoring of surveillance data has the benefit of providing information regarding test performance, and if conducted in real-time, it can enable programs to examine reasons for poor test performance in close proximity to the occurrence. Techniques used in this study could be a valuable addition for postmarketing surveillance of test performance and may become particularly important with the increase in rapid testing methods.

## Introduction

Postmarketing surveillance is conducted to monitor the performance of a pharmaceutical or testing device recently introduced to the marketplace. Because postmarketing surveillance includes data from the general population, it has the ability to detect rare events, which are difficult to detect in pre-licensure studies, such as a clinical trial involving a relatively small number of subjects. In the United States, the Food and Drug Administration (FDA) routinely conducts postmarketing surveillance and has the ability to remove a drug or device from market because of new evidence of problems with efficacy or safety [1]. Postmarketing surveillance has been carried out on rapid testing devices, such as those used to test for the presence of human immunodeficiency virus (HIV) antibody [2], [3]; however, a critique of these types of surveillance activities is that they are often performed retrospectively, once issues with test performance have been repeatedly observed [4]. Here we propose a formal method, routinely utilized in syndromic surveillance [5], that can be used prospectively to improve the sensitivity and timeliness of postmarketing surveillance of screening test performance. This method was developed using data from New York City's sexually transmitted disease (STD) clinics.

**Figure 1:**
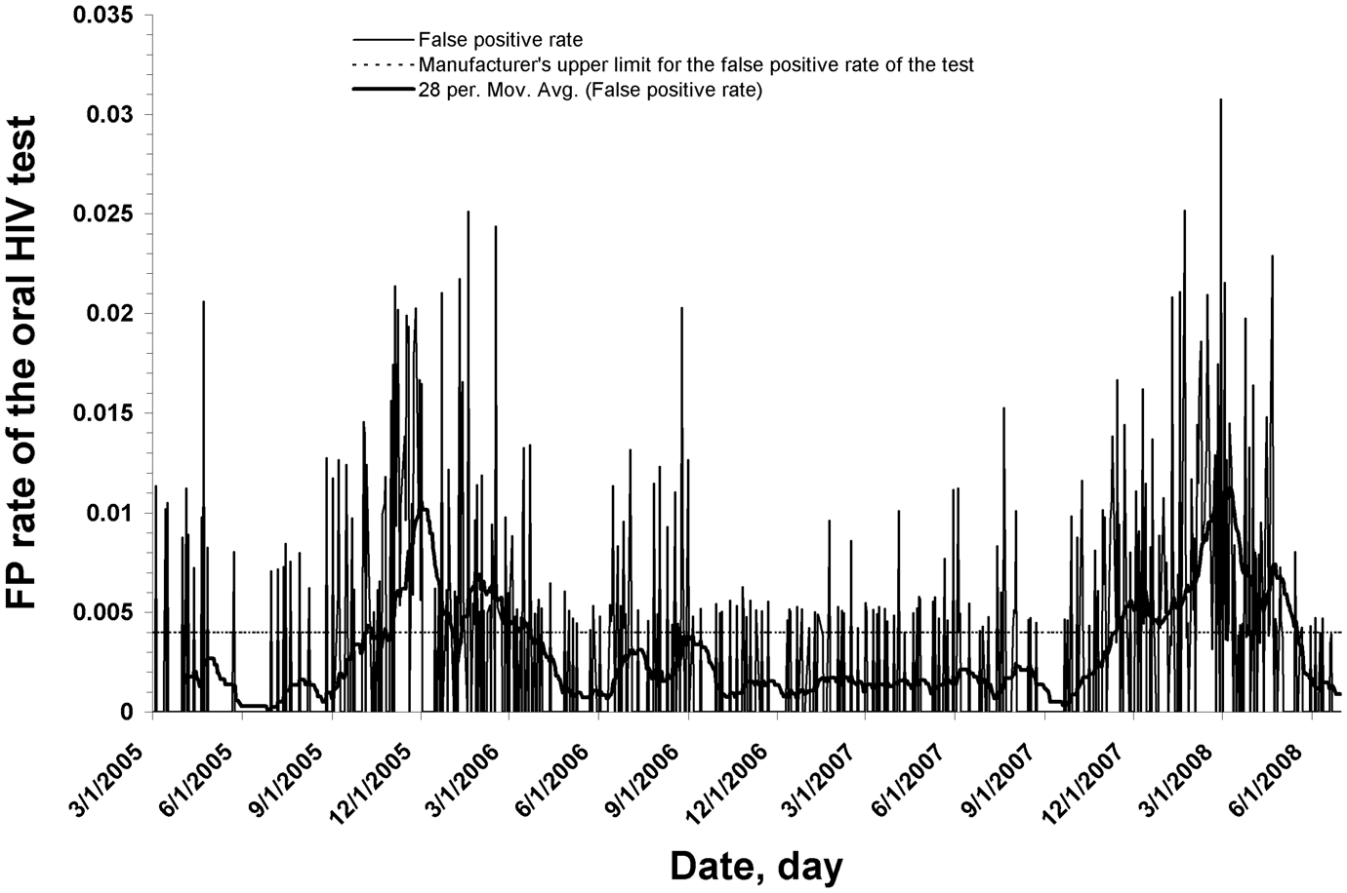
False-positive rate of the rapid HIV test, March 1st 2005 to June 30^th^ 2008. Daily false-positive rate of the oral fluid rapid HIV test, evaluated in New York City's 10 DOHMH STD clinics from March 1st 2005 to June 30^th^ 2008. The moving average was calculated by taking the unweighted average across a 28-day moving window of the FP rate.

In October 2005, the New York City Department of Health and Mental Hygiene (NYC DOHMH) observed unexpectedly high false-positive (FP) rates in the oral fluid-based testing for HIV antibody (OraQuick Advance Rapid HIV-1/2) in several of its 10 STD walk-in clinics. Variations in the false-positive rate had not been previously observed in New York City when whole blood specimens were used with the OraQuick Advance Rapid HIV-1/2 test for HIV antibody. In the United States, all clinical laboratory testing devices are regulated by the Centers for Medicare and Medicaid Services through the Clinical Laboratory Improvement Amendments (CLIA) program. The Orasure oral HIV test used in NYC STD clinics during this time was deemed simple enough to perform to garner a CLIA waiver [6]. As a result, the test was exempt from most CLIA requirements, although the FDA requires facilities to adhere to quality assurance guidelines for CLIA-waved tests to ensure that mistakes are minimized and FDA restrictions for sale of these tests are followed [7]. A review of the specificity (i.e., 1-false positive rate) of the oral test done in 2008 suggested that from 2005 to 2008 there were two distinct peaks in the false-positive (FP) rate, first in late 2005 and again in late 2007 [8] (Figure 1). In fact, from October 2007 to April 2008, the median specificity of the oral test was 99.5%, which is below the manufacturer's claim for the product (95% CI = 99.6–99.9%) (4). This followed similar observations of intermittently low test specificity of the oral HIV test from clinics in other US states, namely Minnesota and California (2, 3). As a result of the issues in 2005, New York City suspended use of the oral HIV test for three weeks in late 2005 and then reintroduced oral fluid-based testing only in conjunction with immediate follow-up rapid whole blood testing after any reactive oral fluid test result (7). When a drop in specificity of the oral HIV test occurred again, in 2008, DOHMH suspended use of oral fluid for HIV-1/2 antibody screening in the spring of 2008, and has since returned to using the same rapid test, but only with whole blood specimens.

In this study, we assessed whether the FP rate of the oral HIV test rose significantly above expectation in each of NYC's STD clinics from 2005 to 2008. Here we defined *expectation* in two ways. In syndromic surveillance an expected value is often calculated using a baseline period in the observed data. Calculation of this expected value tells us what is normally seen in our population. However, it may also be of interest to determine whether the FP rate differs from an acceptable limit, as is often a goal in postmarketing surveillance [1]. Therefore, we attempted to answer both whether the FP rate rose above what was historically experienced in NYC's STD clinics, as well as whether the FP rate rose above the manufacturer's claim of the upper limit of the FP rate for the product. We further assessed whether any increase in the FP rate was related to factors occurring at the clinic level, including operator performance. This method can also be used in real-time, and the ability to conduct prospective postmarketing surveillance could substantially improve the ability to detect issues with performance and the response time to reassess the suitability of such products for the marketplace.

## Methods

Over 170,000 oral fluid-based HIV-1/2 antibody tests were performed in the 10 DOHMH STD clinics in New York City from March 1st, 2005 to June 30th, 2008. Positive rapid oral tests were considered preliminary and were followed up with a confirmatory Western blot (WB) test. Any preliminary-positive test that was found to be WB-negative was deemed to be a false-positive oral test. Data were collapsed by date to calculate a daily FP rate. Information on test date, clinic, and test operator were recorded for each test performed, as well as the manufacturer's product lot number.

SaTScan™ software, v7.0 was applied retrospectively to prospective data collected over the study period to detect temporal clusters in the FP rate of the oral HIV screening test. Data were assumed to be Bernoulli-distributed and significance was assessed through Monte Carlo simulation [9].

To determine whether any observed temporal cluster exceeded the manufacturer's upper limit for the FP rate for the test (0.004), the FP rate was artificially inflated to equal 0.004 over the entire study period. SaTScan was then run on these artificial data, including 999 Monte Carlo simulations. The log likelihood ratio (LLR) for the most likely cluster from the observed data was then ranked among the LLRs of the 999 simulations from the artificial dataset to determine the probability that the observed cluster would have been detected given a FP rate of 0.004. STD clinic was also included as a covariate in a separate scan to determine whether any temporal clusters persisted after adjusting for the effect of clinic. Furthermore, a scan was performed to test the variation by time and clinic simultaneously to assess whether any clinic exhibited a temporal cluster in the FP rate, after adjusting for its own baseline level.

Data were aggregated by test lot for all lots with more than 10 tests. A scan was then performed on these data, stratified by lot, to determine whether any single test lot had a higher than expected FP rate.

Finally, to assess whether the FP rate varied by test operator, a kernel smoothing function was applied to the data aggregated by test operator for all operators who performed at least 100 tests. A weighted linear regression line was also fit to these data to test whether the number of tests performed by an operator was a predictor of the FP rate. The regression line was weighted by the total number of tests performed. Both the kernel smoothing function and regression analysis were performed using R software v2.9 (cran.r-project.org). All data were analyzed anonymously. Neither IRB approval nor informed consents were obtained for this study.

## Results

An average of 181 oral HIV tests were performed daily in the 10 DOHMH STD clinics over the study period (SD = 51.6). The median observed FP rate over this time was 0.003 (Specificity = 99.7%), which is within the manufacturer's claim (99.6–99.9%) for the product. However, two distinct clusters of high FP rates were detected over the study period (**Figure 1**). The largest cluster signal occurred from October 30, 2007 to April 21, 2008 (LLR = 80.5), p = 0.001). The specificity of the oral test during this time was 99.3%, representing a FP rate of 0.007. A second cluster signal was detected when the analysis was limited to dates before the beginning of the first cluster (10/30/07). This second cluster occurred from September 1, 2005 to March 23, 2006 (LLR = 63.8, p = 0.001). The specificity during this time was 99.4% (FP rate  = 0.006).

When comparing the LLR of the first cluster to the 999 simulations in which the data were artificially inflated to have an average FP rate of 0.004, the observed cluster ranked first in Monte Carlo simulation, representing a p-value of 0.001. Furthermore, when comparing the second cluster to the 999 simulations in which the revised baseline period (i.e., 3/1/05 to 10/30/07) was inflated to have an average FP rate of 0.004, the observed cluster again ranked first.

We found the FP rate to vary by clinic (chi-square test of difference, χ^2^ = 82.0, 9df, p<0.001), so the expected FP rate was adjusted for the effect of clinic in the temporal scan. When this was done, the primary cluster period increased in length by ten days, lasting from October 30, 2007 to May 1, 2008 (LLR = 99.8, p = 0.001).

To further investigate whether certain clinics had clusters of high FP rates during the study period, a clinic-level temporal analysis was performed. This analysis produced unique temporal signals in five of the city's ten STD clinics (**Table 1**). All five of these clinic-level signals occurred within either the primary or the secondary citywide temporal signal.

**Table 1:** Clinic-level temporal signals of the daily false positive rate of the Orasure rapid oral HIV test performed in New York City's 10 DOHMH STD clinics from March 1^st^ 2005 to June 30^th^ 2008.

Clinic	Cluster start	Cluster end	LLR	p-value
4	10/22/07	4/16/08	39.046	0.001
7	9/20/05	1/12/06	32.124	0.001
2	2/14/08	4/28/08	21.383	0.001
9	12/3/07	4/21/08	20.887	0.001
1	8/26/05	2/17/06	16.707	0.001

Showing clinic-level signals with a p-value of less than 0.01 only.

There were 94 HIV test kit lots used in NYC during the study period (minimum tests per lot = 10, maximum = 10,614). The test lot-level scan of high FP rates produced six signals, indicating that the FP rate was significantly higher than expectation for six test lots during the study period. Furthermore, all six lot-level signals coincided with either the primary or secondary citywide signal. It is worth noting that all but one of these six test lots were used at multiple STD clinics and the number of FP tests recorded from each lot varied by clinic. For example, one lot was used at nine of the ten clinics with a FP rate of between 0% and 2.4% at each. Finally, test operator was found to be only a marginally significant predictor of the FP rate in the weighted regression analysis (ß = −2.7E-7, p = .055).

## Discussion

Results of our analysis indicate that the temporal scan method was able to detect distinct time periods when the FP rate exceeded both what was usually seen in New York City's STD clinics, as well as what the manufacturer claimed for the product. Monitoring variations in this test's FP rate is important because with rare diseases like HIV, a small decrease in specificity can lead to a large decrease in the positive predictive value (PPV: the proportion of subjects with a positive test who are correctly diagnosed) of a test. For example, a drop in specificity of the oral test from 99.8% to 99.5% translates to a decrease in PPV from 76% to 49% in the New York City STD clinic population. This suggests that when the specificity dropped to 99.5%, more than half of all patients with an HIV-preliminary-positive rapid test result were, in fact, not HIV-positive. When a preliminary-positive screening test is observed, multiple actions are taken that impose a large burden both logistically for the clinic, and more importantly, personally for the patient. Since the NYC DOHMH had instituted the immediate re-testing of preliminary oral positives patients with finger-stick whole-blood tests, the counselors were able to discuss with the patient that the observed discordant tests were most likely due to a false-positive result. However, the additional wait time for the confirmatory HIV test result becomes an added stress for both the patient and the counselors who are delivering this message. Timely action in correcting poor test or operator performance is, therefore, critical to maintaining accurate public health screening.

The two detected clusters correspond well with the observations made by astute NYC DOHMH staff at the time. However, a strength of this scan analysis that cannot be achieved by simple observation is that it compares the observed FP rate pattern to many simulations of the data to see if chance could explain the findings. In this case, it is very unlikely that the existence of these two clusters occurred by chance alone. Thus, our method has the ability to formally test the probability of seeing what was observed in the data in real-time. A further strength of this method is that because it calculates a single test statistic for the most likely cluster in the observed data, it accounts for the multiple testing problems that can arise when many tests are performed at once in a single day at several locations. With this said, our analysis only includes data from New York City, whereas this oral HIV test is performed in clinics across the United States. Therefore, to completely account for issues of multiple testing, data from all test sites during this time period would need to be analyzed concurrently.

The fact that five of the ten STD clinics signaled during the study period indicates that the FP rate in the oral test was not only heterogeneous over time, but also by clinic, or another factor that varied by clinic. These results indicate that including clinic as a covariate improves the sensitivity of the scan analysis to detect clinic-level signals. Identifying clinic-level signals also helps to narrow down the possible location and causes of the observed poor test performance. Two notable factors that may vary by clinic are test lot and test operator. Other studies investigated both test lot and operator as potential causes of variation in the performance of the oral HIV test [2], [3] and found no associations. Our results generally support these studies in finding little evidence that the test's performance varied by operator. However, our analysis does provide evidence that the FP rate varied by test lot. Other unmeasured factors, such as test storage and handling practices or data entry could be causes of the clinic-level variability in test performance; however we were not able to directly measure this. Future testing programs may want to record these types of data in order to include these factors in test surveillance.

We have proposed a simple statistical method of detecting temporal changes in the specificity of a screening test. While this method was applied retrospectively to prospective data, when test results are available electronically, our methods could easily be applied prospectively, which would allow for real-time cluster detection. SaTScan software is free and can be run from within a SAS software session, which can be set-up to run automatically daily, weekly, or at any other time aggregation. A similar temporal scan analysis has been part of New York City's syndromic surveillance activities for several years [10]. As part of this system, SaTScan is used to detect temporal clusters in several unique time series, including ED visits, EMS calls and drug sales. Our method is well suited for use with medical screening, such as mammography, which is known to have low specificity and high variability in radiologist interpretation [11], and could also be used on any screening test that is performed in conjunction with confirmatory testing of diseases such as HIV, syphilis, influenza, and malaria, among others. Furthermore, these methods could be used as a tool in postmarketing surveillance to assess the validity of a manufacturer's claims for their product. Great strides have been made in the development and use of rapid testing, which has helped to reduce the financial and technical barriers in health screening, with particular promise for the developing world. And while the specificity of HIV rapid tests is higher than tests for many other conditions, the performance of these screening tests is often not equal to more rigorous laboratory testing [12], [13] and confirmatory testing remains essential for diagnosis. Developing methods for monitoring the performance of rapid screening tests over time will become particularly important as the use of these tests continues to rise in the future.
